# CRISPR/Cas9-engineered *Drosophila* knock-in models to study VCP diseases

**DOI:** 10.1242/dmm.048603

**Published:** 2021-07-12

**Authors:** Jordan M. Wall, Ankita Basu, Elizabeth R. M. Zunica, Olga S. Dubuisson, Kathryn Pergola, Joshua P. Broussard, John P. Kirwan, Christopher L. Axelrod, Alyssa E. Johnson

**Affiliations:** 1 Louisiana State University, Department of Biological Sciences, Baton Rouge, LA 70803, USA; 2 Integrated Physiology and Molecular Medicine Laboratory, Pennington Biomedical Research Center, Baton Rouge, LA 70808, USA; 3 Department of Translational Services, Pennington Biomedical Research Center, Baton Rouge, LA 70808, USA

**Keywords:** *Drosophila*, IBMPFD, Lysosomes, Ter94, VCP, Mitochondria, MSP-1

## Abstract

Mutations in Valosin Containing Protein (VCP) are associated with several degenerative diseases, including multisystem proteinopathy (MSP-1) and amyotrophic lateral sclerosis. However, patients with VCP mutations vary widely in their pathology and clinical penetrance, making it difficult to devise effective treatment strategies. A deeper understanding of how each mutation affects VCP function could enhance the prediction of clinical outcomes and design of personalized treatment options. The power of a genetically tractable model organism coupled with well-established *in vivo* assays and a relatively short life cycle make *Drosophila* an attractive system to study VCP disease pathogenesis. Using CRISPR/Cas9, we have generated individual *Drosophila* knock-in mutants that include nine hereditary VCP disease mutations. Our models display many hallmarks of VCP-mediated degeneration, including progressive decline in mobility, protein aggregate accumulation and defects in lysosomal and mitochondrial function. We also made some novel and unexpected findings, including nuclear morphology defects and sex-specific phenotypic differences in several mutants. Taken together, the *Drosophila* VCP disease models generated in this study will be useful for studying the etiology of individual VCP patient mutations and testing potential genetic and/or pharmacological therapies.

## Abstract (precis)

**Summary:** We generated a collection of nine *Drosophila* knock-in models that mimic VCP-related diseases and revealed novel pathologies, including sex-dependent effects and laminopathies.

## INTRODUCTION

Multisystem proteinopathy (MSP-1) is an autosomal dominant disease characterized by adult onset degeneration of muscles, bones and neurons (Kimonis et al., 2008b; Weihl et al., 2009; Ju and Weihl, 2010). The presenting symptom in the majority of patients (∼90%) is muscle weakness, which becomes outwardly apparent around midlife (Kimonis et al., 2008b; Al-Obeidi et al., 2018). Approximately 40% of patients are affected by Paget's disease of the bone (PDB) and exhibit hip or spine pain, tenderness in their bones and/or cranial bone deformities. Approximately one-third of patients also develop frontotemporal dementia (FTD), characterized by prominent behavior and language dysfunction, difficulties in preserving memories and loss of social awareness (Kimonis et al., 2008b; Al-Obeidi et al., 2018). In addition to these three degenerative conditions, amyotrophic lateral sclerosis (ALS) and Parkinson's disease are linked to VCP mutations (Johnson et al., 2010; Spina et al., 2013; Benatar et al., 2013; Regensburger et al., 2017; Al-Obeidi et al., 2018). To date, PDB and Parkinson's disease are the only conditions with any viable treatment options that can improve pathology in some cases (Merlotti et al., 2009; Chan et al., 2012).

A seminal molecular genetic study revealed that MSP-1 results from missense mutations in the gene encoding valosin containing protein (VCP) (Watts et al., 2004), and over 30 missense mutations in VCP have now been identified (Weihl et al., 2009). VCP (p97 in mouse, Ter94 in *Drosophila* and CDC48 in yeast) is a highly conserved Type II AAA+ ATPase that is essential for cell and organismal survival (Moir et al., 1982; Müller et al., 2007), and participates in diverse cellular activities (Baek et al., 2013). VCP is 97 kDa in size and consists of three domains: the N-terminal domain (N) and two ATPase domains (D1 and D2). The N-terminal domain binds substrates and co-factors to regulate VCP subcellular distribution and substrate specificity (Buchberger et al., 2015). The two ATPase domains (D1 and D2) confer catalytic activity and promote hexamerization of VCP monomers (Wang et al., 2004). Disease mutations are mostly clustered in the N-terminal and D1 domains, and are all autosomal dominant (Watts et al., 2004; Al-Obeidi et al., 2018).

A critical function of VCP is to maintain protein and organelle homeostasis in the cell (Nalbandian et al., 2011; Baek et al., 2013; van den Boom and Meyer, 2018). Accordingly, muscle biopsies from MSP-1 patients commonly exhibit inclusions of ubiquitin and TDP-43 (Kimonis et al., 2008b; Weihl et al., 2009). VCP has essential roles in four major cellular homoestasis pathways: the ubiquitin-proteasome system (UPS); endoplasmic reticulum-associated degradation (ERAD); DNA damage response; and the autophagy-lysosome system (Wójcik et al., 2006; Krick et al., 2010; Chou et al., 2011; Nalbandian et al., 2011; Meyer et al., 2012; Ramadan, 2012; Dantuma et al., 2014; Singh et al., 2019). However, pathogenic mutations seem to primarily affect the autophagy-lysosome system (Ju et al., 2009; Tresse et al., 2010; Ju and Weihl, 2010; Chang et al., 2011; Johnson et al., 2021). Notably, overexpression of VCP disease mutations disrupt the integrity of a tubular lysosomal network in *Drosophila* muscles, leading to the accumulation of cargo-loaded autophagosomes in the cytoplasm (Johnson et al., 2015). VCP is also essential for autophagy-dependent turnover of lysosomes (‘lysophagy’) and mitochondria (‘mitophagy’) (Tanaka et al., 2010; Papadopoulos et al., 2017). Consequently, damaged lysosomes and mitochondria accumulate in tissues expressing VCP disease mutations (Kim et al., 2013; Papadopoulos et al., 2017; Zhang et al., 2017). Thus, a failure of VCP-dependent proteostasis mechanisms, particularly in the autophagy-lysosome system, is likely a major driver of the disease pathogenesis.

A confounding aspect of VCP diseases is that patients with VCP mutations exhibit an array of seemingly unpredictable phenotypes. A large genotype-phenotype study of 231 individuals harboring 15 distinct VCP mutations did not identify any striking correlations between mutation and clinical features; patients with identical VCP mutations even exhibited different clinical symptoms (Al-Obeidi et al., 2018). Thus, VCP mutations exhibit a varying degree of phenotypic penetrance that could be further complicated by interactions with other genetic and/or environmental factors. Having an experimental genetic model system to study the etiology of individual patient mutations in various biological and/or environmental contexts could shed light on molecular aspects of the disease that cannot be controlled in a clinical setting. Moreover, results from such studies could improve personalized treatment options and patient outcomes.

Using CRISPR gene editing tools, we have generated nine knock-in VCP mutants in *Drosophila melanogaster*. Our *Drosophila* knock-in models offer the advantage of *in vivo* study of each VCP mutant in a genetically tractable model organism with a relatively short life cycle. Moreover, each mutant is a gene replacement of the endogenous VCP gene, which more precisely mimics the genetic disease in human patients compared to overexpression models. Importantly, we validate that our models display progressive degenerative phenotypes and proteinopathies that mirror MSP-1. On a cellular level, many of the mutations display defects in lysosomal and/or mitochondrial function. Overall, we have established a set of VCP disease models that can be used to dissect underlying cellular defects caused by VCP mutations, and provide a time and cost-effective model to test genetic and/or chemical therapies.

## RESULTS

### Generation of VCP disease models using CRISPR/Cas9-targeted gene replacements

**Fig. 1.**
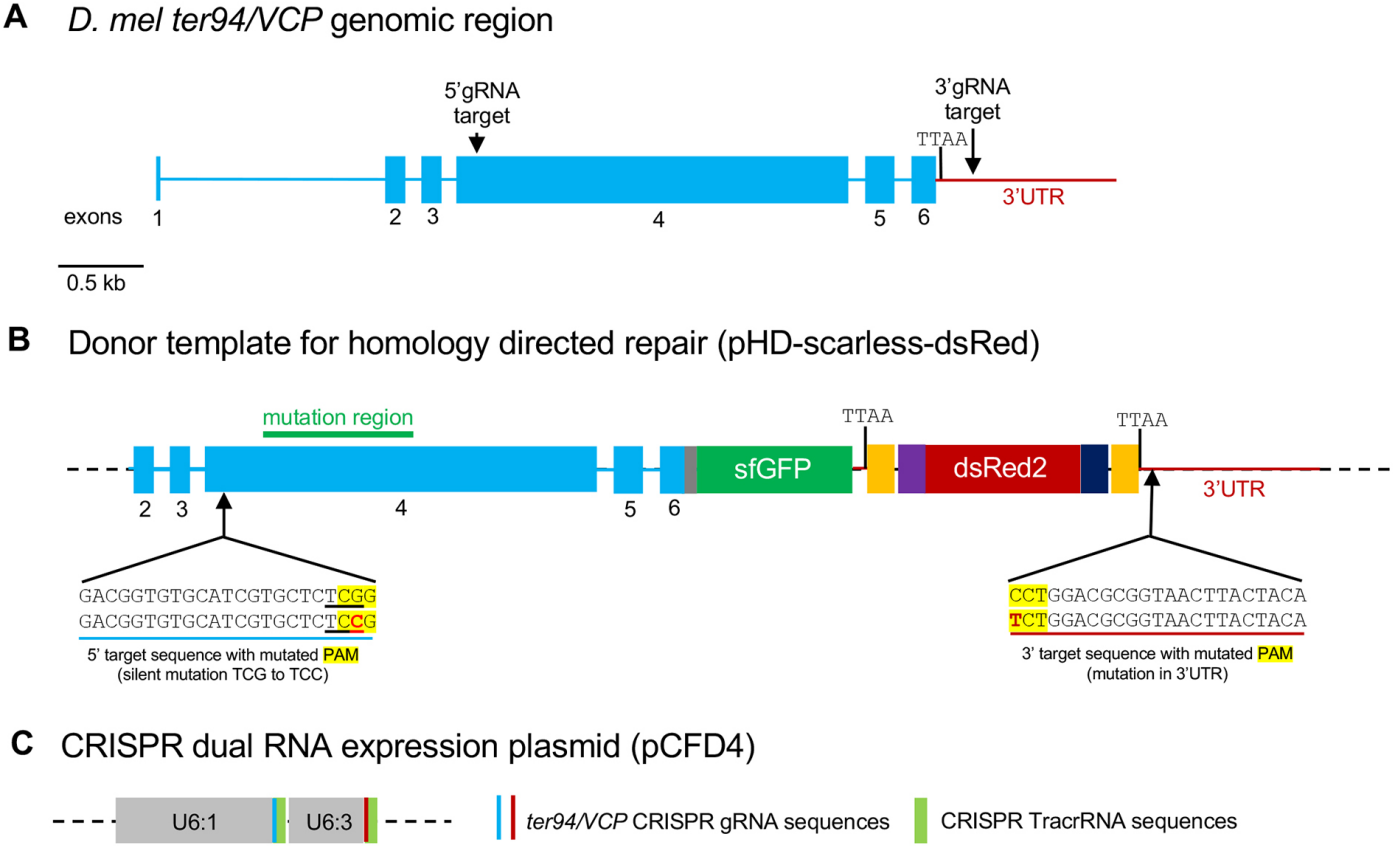
**VCP mutant CRISPR knock-in strategy.** (A) *Drosophila Ter94/VCP* genomic region. (B) Schematic of the donor template that was used for homology-directed repair to generate VCP mutant knock-ins. (C) Schematic of the dual expression plasmid that was used to co-express both 5′ and 3′ tracrRNA-gRNA fusions.

**Fig. 2.**
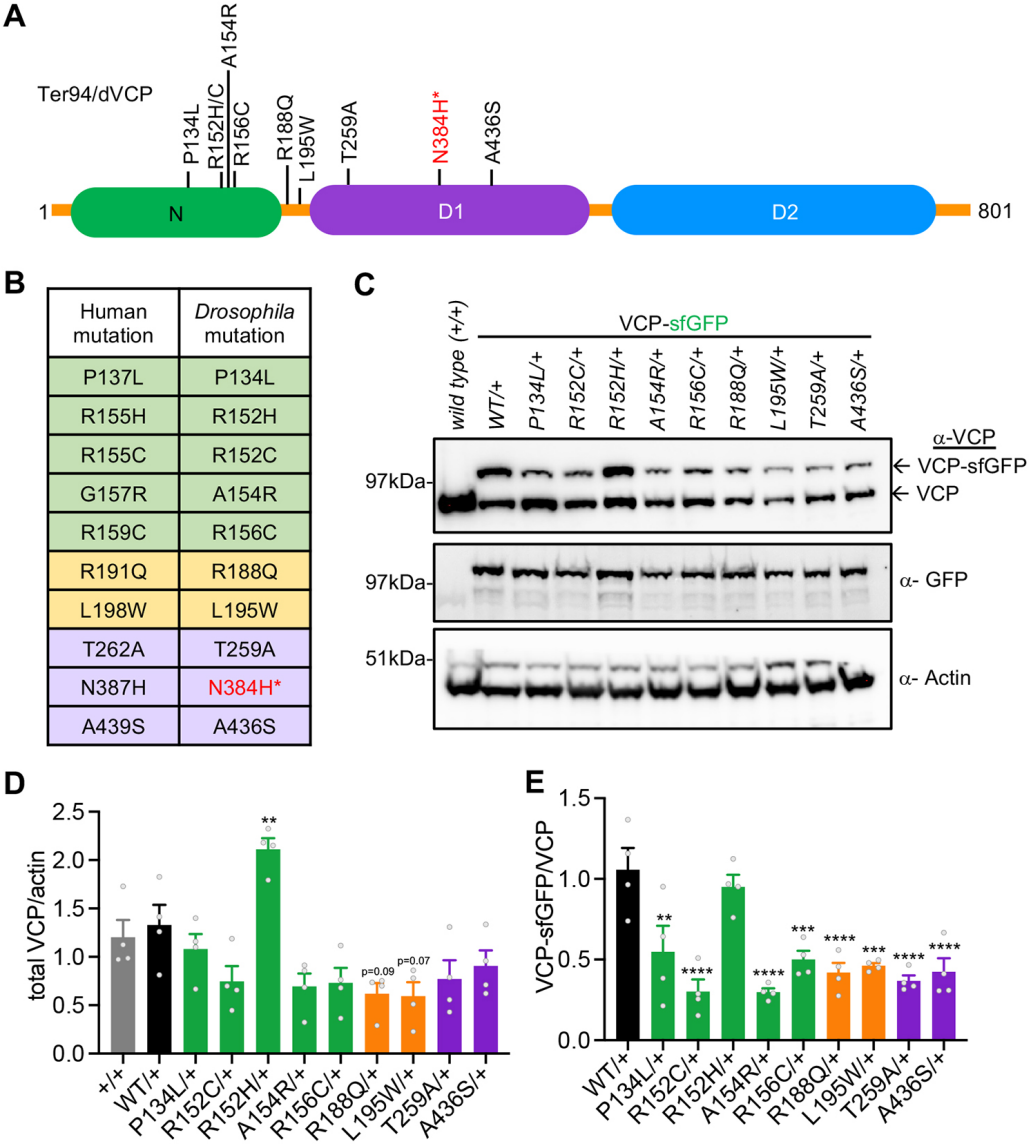
**VCP mutant expression.** (A) Schematic of VCP protein with the relative position of pathogenic mutations. *N384H was embryonic lethal and was not studied further. (B) Table of human and homologous *Drosophila* VCP mutations. (C) Representative western blot of VCP heterozygous mutant expression levels. (D) Quantification of total VCP protein abundance. (E) Quantification of wild-type versus mutant VCP protein levels. Data are mean±s.e.m. *n*=4 independent biological replicates. **P<0.01, ***P<0.001, ****P<0.0001 (one-way ANOVA with Dunnett's multiple comparisons).

To generate VCP disease models, we used CRISPR/Cas9 gene editing to introduce VCP mutations at the endogenous *Drosophila Ter94/dVCP* locus, hereafter referred to as VCP (Fig. 1A). The *VCP* gene includes six exons, and all ten pathogenic mutations selected in this study (Fig. 2B) reside in exon 4, the largest exon in the *VCP* gene (Fig. 1A,B). The fly CRISPR target finder online program (https://flycrispr.org/target-finder/) was used to select optimal guide RNA target sites (Gratz et al., 2014). An optimal 5′ guide RNA target site was selected upstream of the most N-terminal mutation so that the same base plasmid could be mutagenized to generate all ten individual mutant plasmids (Fig. 1). An optimal 3′ guide RNA target site was selected downstream of the first endogenous TTAA site in the 3′UTR (Fig. 1).

The donor template was generated by subcloning *VCP* genomic regions into the pHD-ScarlessDsRed plasmid, which contains a 3xP3-DsRed expression cassette flanked by TTAA-PBac transposon ends (Fig. 1B). The DsRed expression cassette serves as a selectable marker and the TTAA-PBac transposons allow for ‘scarless’ removal of the cassette after CRISPR/Cas9 gene editing if desired (Gratz et al., 2015a,b). A C-terminal superfold GFP (sfGFP) tag was also incorporated before the stop codon of the VCP coding region to enable endogenous localization studies. Approximately 1 kb of homologous genomic regions outside of the guide RNA target sites were included to promote homology-directed repair, and the protospacer adjacent motif (PAM) sequences were mutated in the donor plasmid to prevent re-excision. The 5′ and 3′ gRNA target sequences were simultaneously cloned into the dual expression pCFD4 plasmid, which contains the U6:1 and U6:3 promoters, and CRISPR tracrRNA sequences (Fig. 1C; Port et al., 2014). The donor plasmid and guide RNA expression plasmid were co-injected into germline expressing Cas9 embryos (vasa>cas9). Transgenic animals were first selected by fluorescent DsRed expression and then validated by sequencing (Fig. S1).

### Expression of pathogenic VCP mutations in *Drosophila* models

We selected ten hereditary pathogenic VCP mutations that were also conserved in the *Drosophila VCP* gene (Fig. 2A,B). We were able to successfully generate nine of the ten heterozygous mutants; however, the N384H mutant failed to produce live transgenic animals after multiple attempts, suggesting that expression of even a single copy of this mutant in *Drosophila* may be embryonic lethal. Additionally, we were unable to generate homozygous adult animals of the other nine mutants, although homozygous wild-type VCP tagged with sfGFP were able to survive to adulthood. Thus, VCP mutants are homozygous lethal, consistent with the autosomal dominant nature of VCP diseases (Watts et al., 2004). Consequently, all of our VCP mutant models are heterozygous for the disease mutations.

We then examined expression of the mutant VCP proteins. Each of the mutants were tagged with an sfGFP tag, which allowed us to resolve the mutant VCP protein from wild-type VCP protein by denatured gel electrophoresis. Each of the VCP mutants expressed both wild-type and sfGFP-tagged mutant proteins as expected (Fig. 2C). Total VCP protein was unaltered in most of the mutants, with the exception of the R152H mutant, which had significantly more total VCP protein on average (Fig. 2C,D). We then compared the levels of wild-type versus mutant VCP protein. In most of the mutant animals, wild-type protein was more abundant compared to mutant VCP protein. Again, R152H was the exception, displaying equal levels of both wild-type and mutant VCP protein (Fig. 2E). Although we cannot rule out differences in gene expression, the fact that the mutants are being expressed from the endogenous chromosome would suggest that the differences in VCP protein abundance is due to differences in protein stability. Taken together, we generated nine VCP disease mutant knock-in models that can be used to study aspects of VCP disease on a molecular, cellular and organismal level.

### VCP disease mutations alter locomotor capability in an age- and sex-dependent manner

**Fig. 3.**
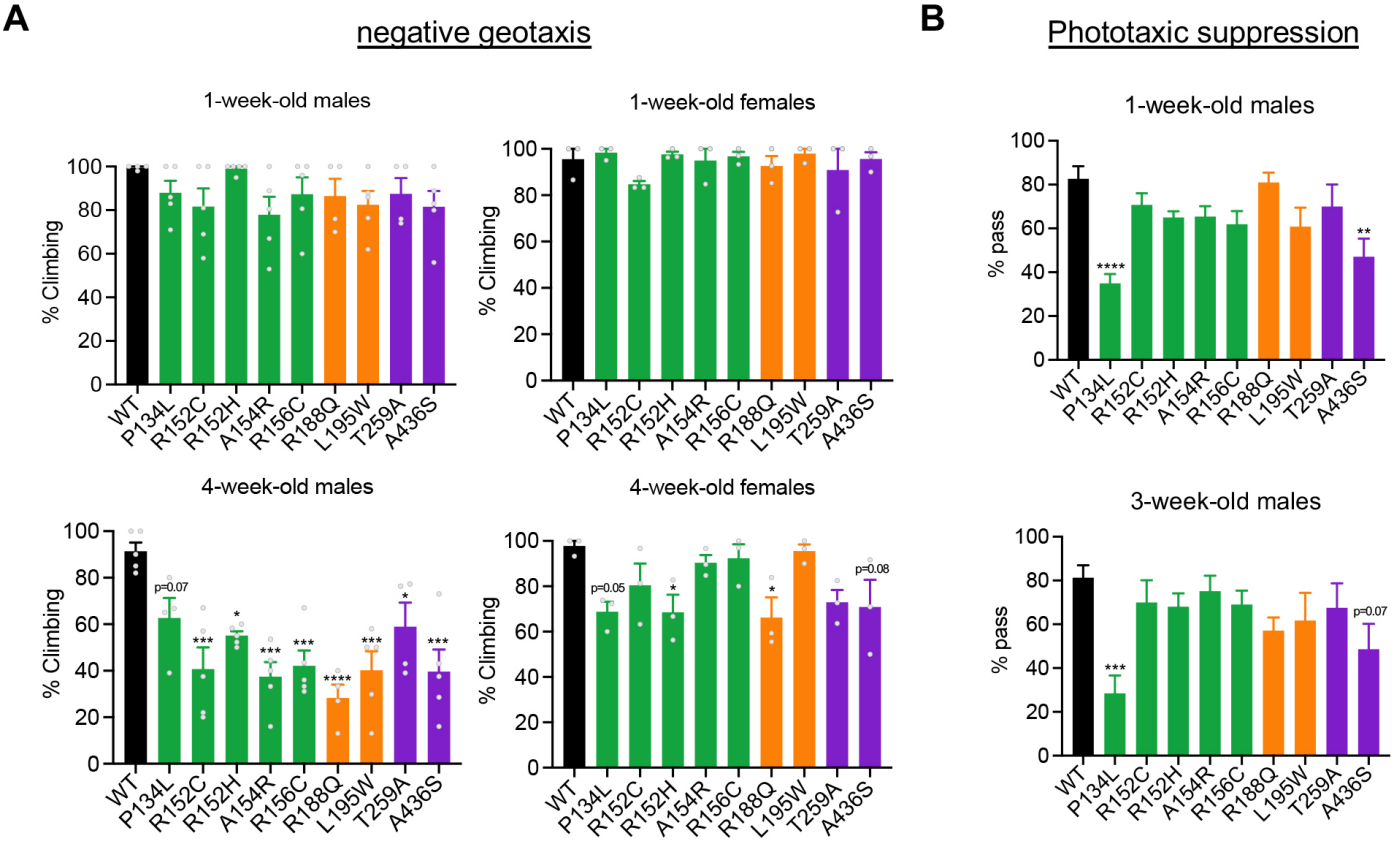
**Mobility and learning/memory analyses of VCP disease models.** (A) Negative geotaxis assay of the indicated genotypes in males and females at 1 week and 4 weeks of age. *n*=4 independent biological replicates with >45 flies/replicate. (B) Phototaxic suppression assay for the indicated genotypes. Male flies were assessed at 1 week and 3 weeks of age. Data are mean±s.e.m. *n*=10. *P<0.05, **P<0.01, ***P<0.001, ****P<0.0001 (one-way ANOVA with Dunnett's multiple comparisons). WT, wild type.

The presenting symptom in most MSP-1 patients is muscle weakness that results in visible locomotor deficits (Kimonis et al., 2008b; Weihl et al., 2009). Patients start showing symptoms during early to late midlife, which progressively worsen with age. To verify whether our models mimic this phenotype, we performed negative geotaxis assays to examine the locomotor ability of the VCP mutants. Because the disease is progressive, we examined the climbing ability of each mutant 1 week post eclosion (young) and 4 weeks post eclosion (midlife) in both male and female flies. At 1 week, we observed no significant differences among the mutants compared to VCP-WT animals in either sex (Fig. 3A). However, at 4 weeks of age, significant mobility impairments became apparent, but in a sex-specific manner. In male flies, the majority of the mutants exhibited significant mobility impairment; however, in females, only two genotypes (R152H and R188Q) displayed significant mobility impairments (Fig. 3A). Thus, our VCP disease models exhibit both age- and sex-dependent motor impairments.

### P134L and A436S mutations are accompanied by learning/memory impairments

Approximately a third of patients with MSP-1 are affected with FTD, which comprises loss of memory and other cognitive functions (Kimonis et al., 2008a; Al-Obeidi et al., 2018). To examine whether any of our disease models displayed cognitive impairments, we conducted an aversive phototaxis suppression assay, a common assay to test learning and memory in flies (see Materials and Methods). Because memory impairments are also progressive in MSP-1, we tested memory at 1 week (young) and 3 weeks (midlife) of age. Male flies were tested in this assay as they were more strongly affected by VCP mutations in the climbing assay (Fig. 3A). At 1 week old, we observed a significant impairment in short-term memory in two of the nine mutants (P134L and A436S; Fig. 3B). At 3 weeks, P134L continued to display significant short-term memory impairments, and A436S trended toward a significant impairment (*P*=0.07) (Fig. 3B). We did not observe significant memory impairments in the remaining seven mutants at either 1 or 3 weeks of age. Thus, P134L and A436S mutants displayed learning/memory impairments irrespective of age.

### R152C, R152H, L194W and T259A mutants exhibit reduced lifespan

**Fig. 4.**
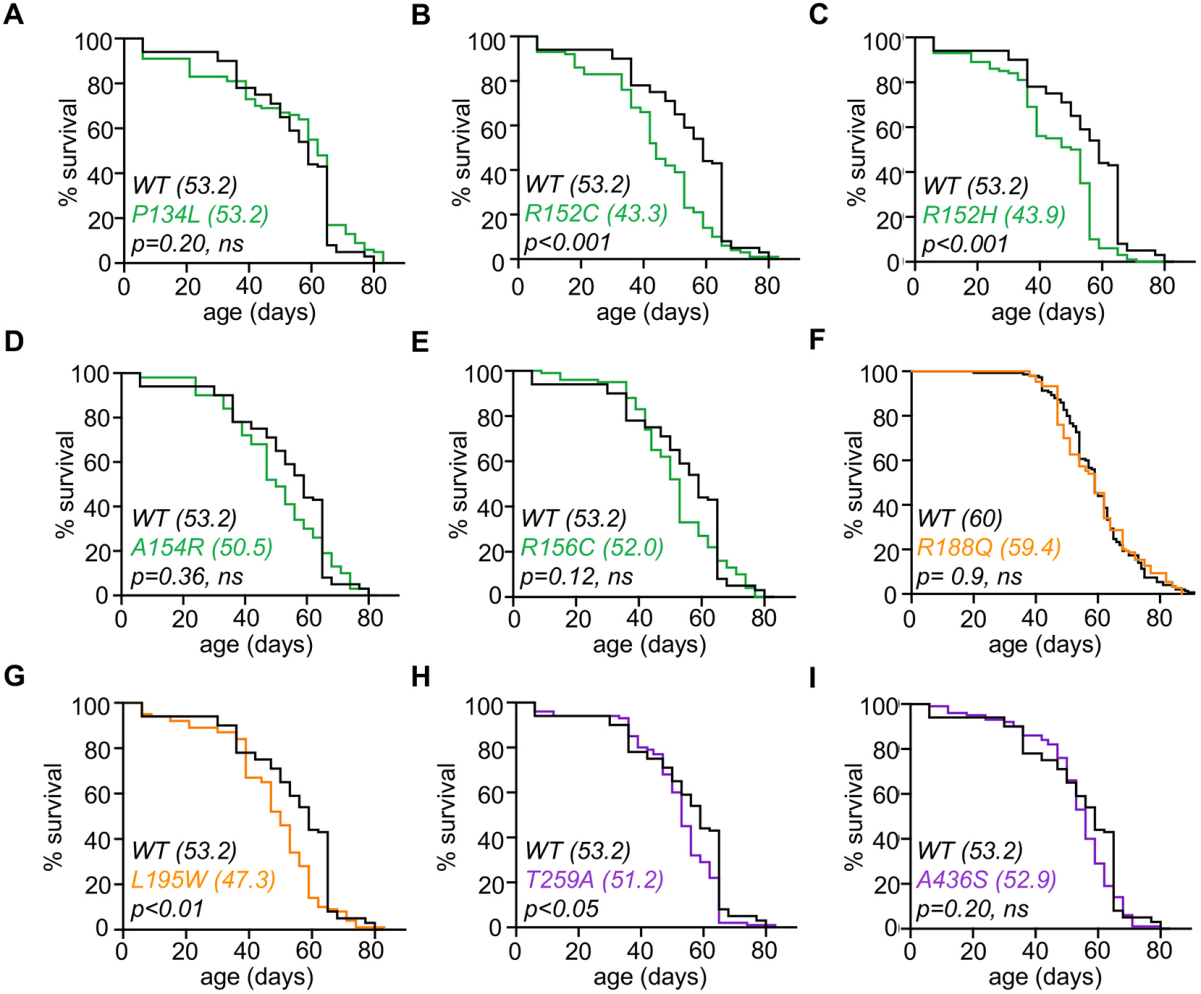
**Lifespan analysis of VCP disease models.** (A-I) Lifespan assay of the genotypes indicated. Median lifespans are presented in parenthesis on each graph. A log-rank test was used to determine significance, *P*-values are indicated on each plot. *n*>90 for each genotype. WT, wild type.

Given the age-related locomotory defects, we investigated whether there was any effect on lifespan. Most VCP mutants did not display a significant reduction in lifespan compared to VCP-WT animals (Fig. 4A-I). However, four mutants (R152C, R152H, L195W and T259A) displayed a modest but significant reduction in lifespan (Fig. 4B,C,G,H). Collectively, these results are consistent with MSP-1, in which the majority of patients exhibit significant motor impairments at midlife but can still live well into their 70s (Kimonis et al., 2008a).

### Disease mutations display multiple proteostasis pathologies

**Fig. 5.**
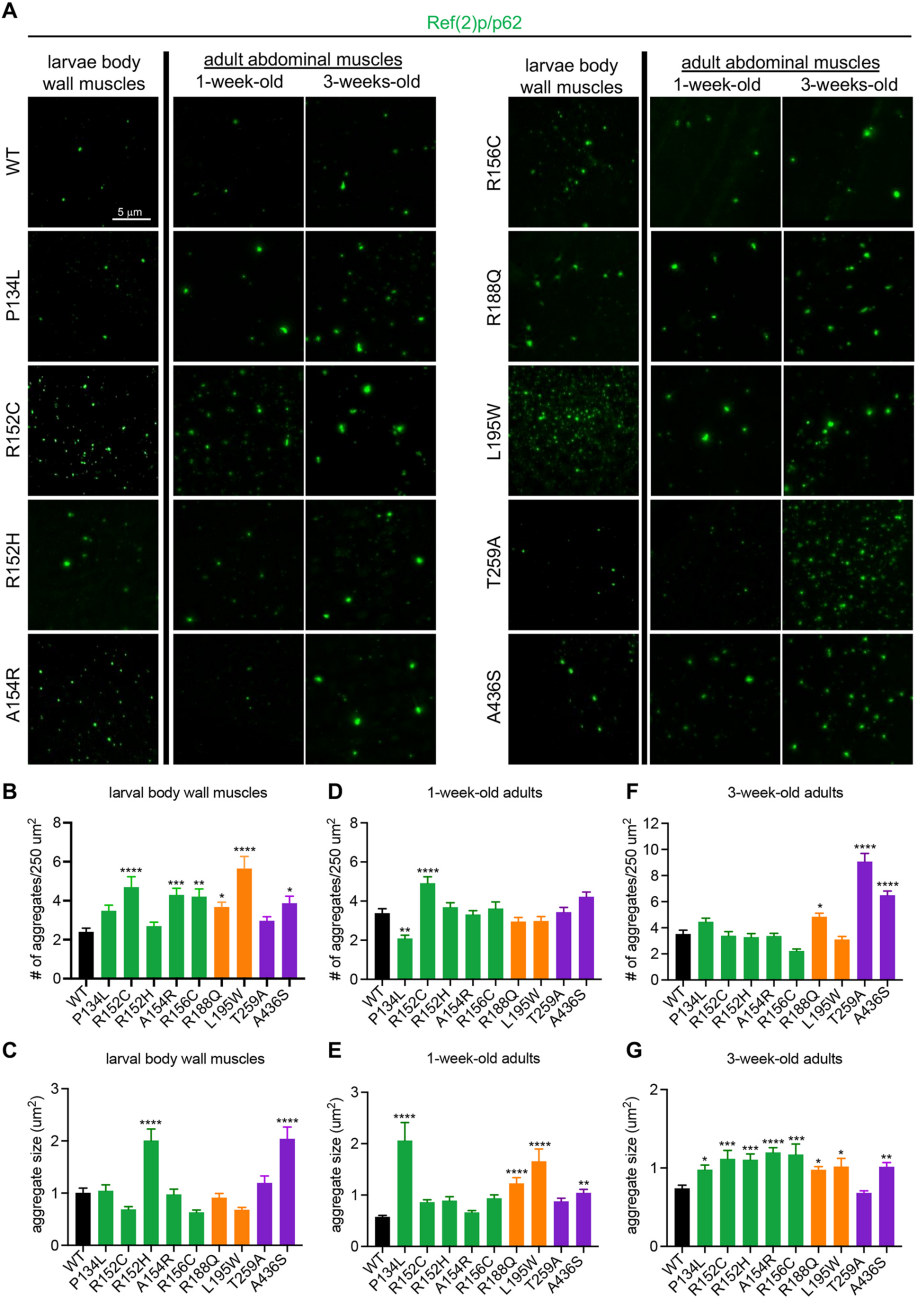
**Proteostasis analysis in VCP disease models.** (A) Representative images of p62 staining in larval body wall muscles and adult abdominal muscles at 1 week and 3 weeks of age in the indicated genotypes. (B,C) Quantification of the number (B) and size (C) of aggregates observed in larval body wall muscles. *n*=4 independent animals. (D,E) Quantification of the number (D) and size (E) of aggregates observed in 1-week-old adult abdominal muscles for the indicated genotypes. (F,G) Quantification of the number (D) and size (E) of aggregates observed in 3-week-old adult abdominal muscles for the indicated genotypes. Data are mean±s.e.m. For number of aggregates, *n*>50 muscles from four independent animals. For aggregate size, *n*>100 aggregates from four independent animals. *P<0.05, **P<0.01, ***P<0.001, ****P<0.0001 (one-way ANOVA with Dunnett's multiple comparisons). WT, wild type.

VCP diseases are associated with widespread proteostasis defects, particularly in the autophagy-lysosome system (Ju et al., 2009). Thus, we assessed various parameters of proteostasis defects at different stages in the VCP disease models. First, we examined protein aggregates by staining muscles with an antibody against Ref(2)p/p62, a protein that specifically marks aggregates for autophagic degradation (Nezis et al., 2008). In larval muscles, we found that all but two mutants (P134L and T259A) displayed some degree of p62 pathology, including increased p62^+^ aggregate abundance and/or aggregate size compared to wild type (Fig. 5A-C). In adult abdominal muscles, similar p62^+^ aggregate pathologies were apparent and worsened with age in the majority of the mutants (Fig. 5A,D-G).

**Fig. 6.**
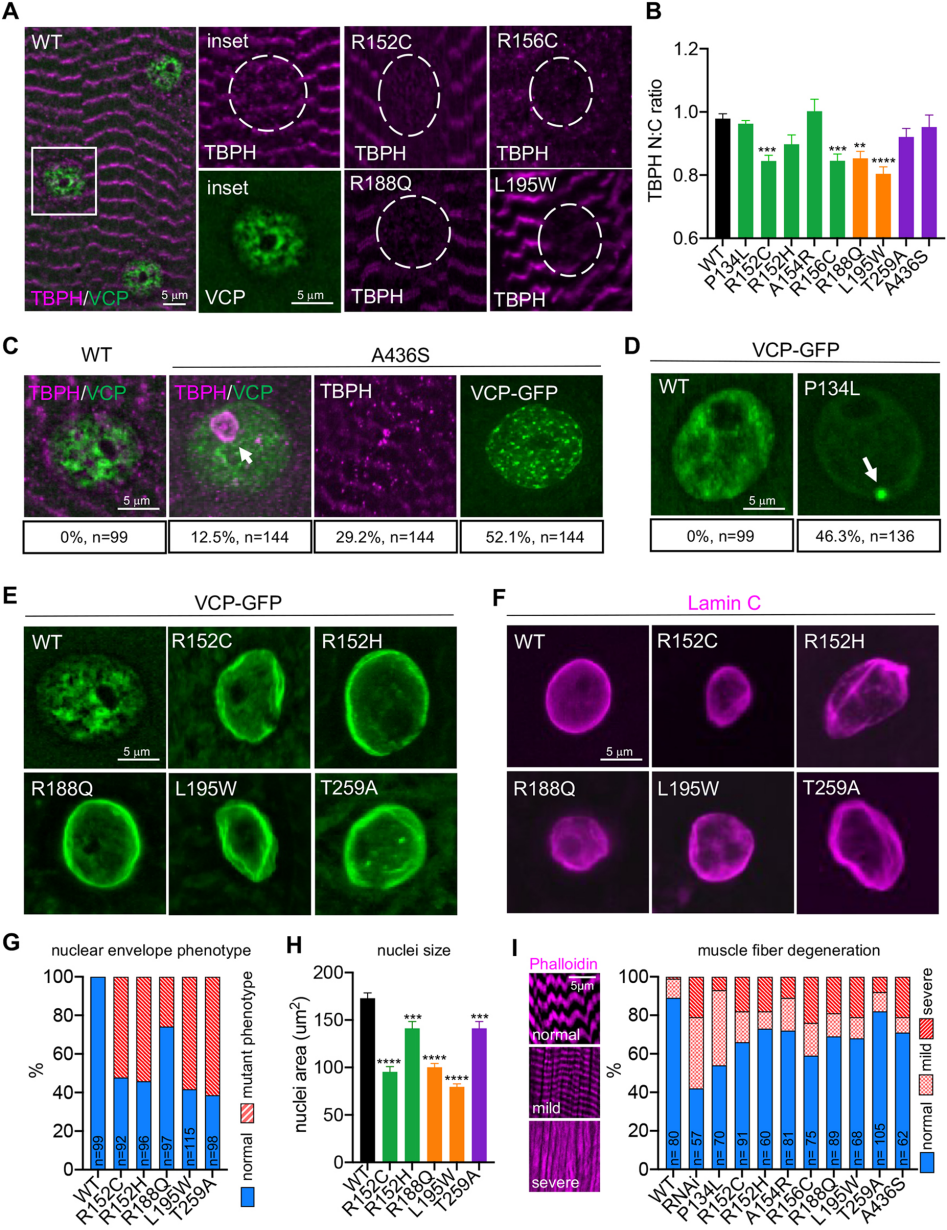
**Cellular pathology in larval muscles.** (A) TBPH staining in larval body wall muscles for the indicated genotypes. Dashed circles indicate the nucleus. (B) Nuclear to cytoplasmic ratio of TDP43 distribution in larval body wall muscles for the indicated genotypes. *n*>50 nuclei from four independent animals. (C) TBPH and VCP mutant localization in A436S mutants (scoring is indicated below the representative images; *n* represents independent nuclei or muscles from four independent animals). Arrow indicates TBPH accumulation in the nucleolus. (D) VCP localization in wild type (WT) and P134L mutants (scoring is indicated below the representative images; *n* represents individual nuclei from four independent animals). Arrow indicates VCP aggregate. (E,F) VCP localization (E) and Lamin C staining (F) in the indicated genotypes. (G) Quantification of the percentage of nuclei displaying the nuclear envelope phenotype (*n* is indicated on the individual bars and represents individual nuclei scored from four independent animals). (H) Quantification of nuclei size for the indicated genotypes (*n*>50 nuclei from four independent animals). (I) Phalloidin staining for larval muscles displaying different degrees of defects, and quantification of the percentage of healthy, mild and severely defective muscles (*n* is indicated on the individual bars and represents individual muscles scored from five independent animals). Data are mean±s.e.m. **P<0.01, ***P<0.001, ****P<0.0001 (one-way ANOVA with Dunnett's multiple comparisons).

TDP-43 cytoplasmic accumulation is also a hallmark of VCP muscle pathologies (Ritson et al., 2010). Thus, we examined potential TDP-43 pathologies by staining muscles with an antibody against the *Drosophila* TDP-43 ortholog, TBPH. We examined larval muscles because proteostasis defects were already apparent at this early stage (Fig. 5A-C). In wild-type muscles, TBPH signal was visible on the sarcomeres, as well as diffusely in the nucleus (Fig. 6A). This staining pattern is consistent with previous reports of TBPH localization in *Drosophila* adult muscles (Llamusi et al., 2013). In four of the mutants (R152C, R156C, R188Q and L195W), we observed significant redistribution of TBPH from the nucleus to the cytoplasm (Fig. 6A,B). Intriguingly, the A436S mutant displayed an array of atypical TBPH localizations; in 12.5% of nuclei, TBPH accumulated inside the nucleolus and in 30% of cells, TBPH formed large cytoplasmic inclusions (Fig. 6C).

Finally, we examined VCP mutant localization as mutant VCP proteins have also been found to be mislocalized in disease tissue samples (Weihl et al., 2009). Each VCP knock-in mutant is tagged with an sfGFP tag, which enabled us to directly examine VCP mutant localization. Similar to previous reports, we found that endogenously expressed VCP-GFP localizes diffusely in the cytoplasm and in discrete structures around the nucleus (Johnson et al., 2021). In the VCP mutants, we again noted a wide range of atypical phenotypes. In two of the mutants, VCP aggregated around the nucleus in either a dispersed pattern (A436S) or as a large single VCP aggregate (P134L) (Fig. 6C,D). But the most prominent and striking phenotype in the majority of the mutants was an ectopic concentration of VCP around the nuclear periphery in a concentric pattern reminiscent of nuclear Lamins (Nmezi et al., 2019) (Fig. 6E). To examine this further, we stained muscles with a Lamin C antibody. Strikingly, we found that in five of the mutants, the nuclear lamina was disorganized in a significant proportion of cells (Fig. 6F,G). Moreover, the nuclei in these mutants were on average smaller compared to wild type (Fig. 6H). Notably, similar defects in nuclear lamina structure and nuclear size are associated with the accelerated aging disease Hutchinson–Gilford progeria syndrome (HGPS; Taimen et al., 2009). Thus, disruption of nuclear architecture could also be a contributing factor to VCP disease progression (see Discussion). Finally, we stained muscles with Phalloidin to assay overall muscle integrity. Consistent with the array of cellular defects observed, all of the mutants displayed signs of muscle fiber degeneration (Fig. 6I).

### Disease mutations in VCP disrupt tubular lysosomal networks in muscle cells

**Fig. 7.**
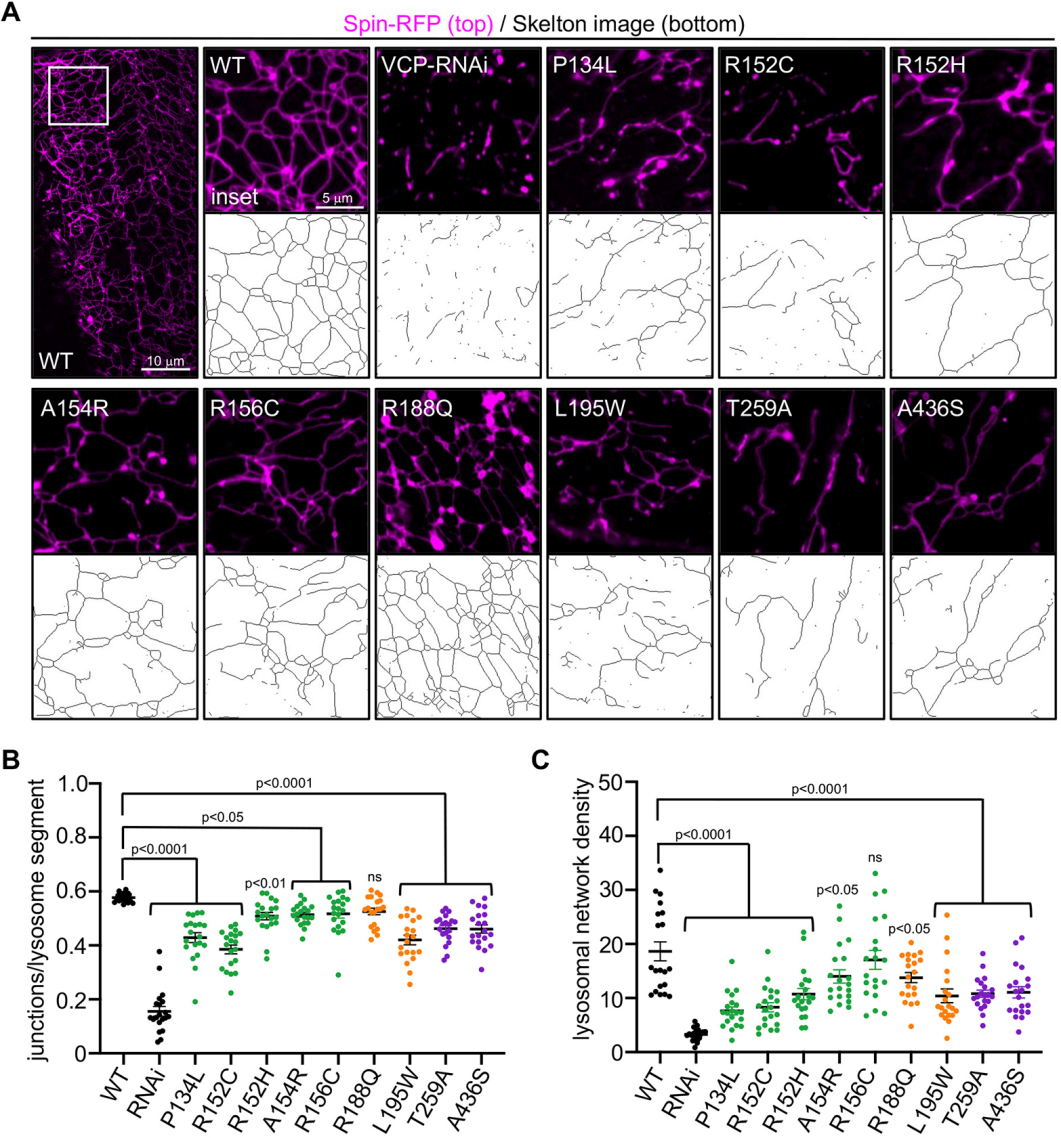
**Tubular lysosomal network analysis in VCP disease models.** (A) Representative images of lysosomes in the third instar larval body wall muscle of the indicated genotypes. Below each image is a representative skeleton image obtained using the Skeletonize plug-in in ImageJ for each of the indicated genotypes. (B,C) Quantification of network integrity (number of junctions/object) (B) and density (objects/µm^2^) (C) for the genotypes indicated. Data are mean±s.e.m. Statistical significance was determined using one-way ANOVA with Dunnett's multiple comparisons (*P*-values are indicated on the graphs, *n*=20 muscles from four independent animals). ns, not significant.

Previously, we found that VCP is required for the maintenance and integrity of a tubular lysosomal network in the muscle cells of *Drosophila* (Johnson et al., 2015). Disruption of the tubular network correlates with progressive degenerative phenotypes that mirror VCP-related diseases, suggesting that tubular lysosome disruption could contribute to VCP disease pathology (Johnson et al., 2021). Thus, we examined the effect on the lysosomal lattice in our VCP disease models by imaging RFP-tagged Spinster, a lysosomal membrane protein (Sweeney and Davis, 2002). In wild-type animals, we observed a compact and dynamic network consistent with previous findings (Fig. 7A; Johnson et al., 2015). In contrast, we observed varying degrees of lysosomal defects in the muscles of several mutants (Fig. 7A). To quantify the varying penetrance, we scored two parameters. First, we quantified the number of junctions per lysosome segment as a measure of network integrity. Second, we quantified the integrated intensity per 100 μm^2^ area as a measure of network density. Consistent with our previous findings (Johnson et al., 2015), inhibition of VCP by RNAi displayed severe defects for both parameters measured (Fig. 7B,C). All of the mutants, except R188Q, displayed significant defects in network integrity (Fig. 7A,B). Similarly, all mutants displayed significant decreases in network density, with the exception of R156C (Fig. 7A,C). Collectively, we observed that most of the VCP mutants displayed lysosomal defects, albeit with varying levels of penetrance.

### Substrate and sex-specific defects in mitochondrial respiratory function in VCP mutants

**Fig. 8.**
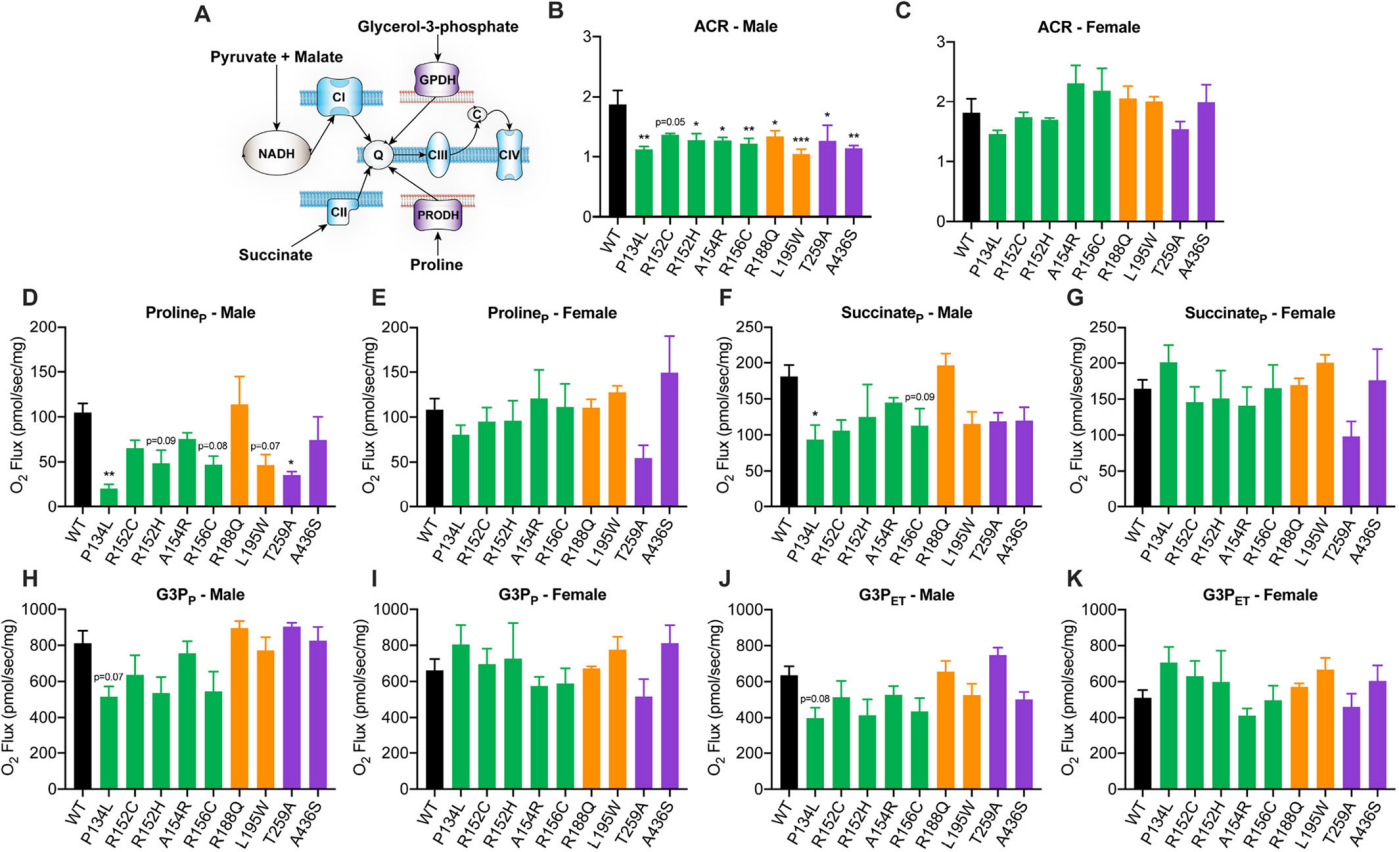
**Mitochondrial respiratory function in VCP mutants.** (A) Schematic illustration of substrate coupling to mitochondrial respiratory pathways evaluated by high-resolution respirometry. (B-K) Acceptor control ratio (ACR) (B,C) and respiration supported by proline (D,E), succinate (F,G) and glycerol-3-phosphate (G3P) in the presence of ADP (P) (H-I) and FCCP (electron transfer) (J-K) in aged male or female wild type (WT) and VCP mutants. Data are mean±s.e.m. *n*=4 independent biological replicates with three flies/replicate. *P<0.05, **P<0.01, ***P<0.01 (one-way ANOVA with Dunnett's multiple comparisons). P, OXHPOS; ET, electron transfer.

Cells expressing pathogenic VCP mutations exhibit reduced ATP levels (Chang et al., 2011; Bartolome et al., 2013), perhaps due to compromised mitochondrial respiration and/or failure to clear damaged mitochondria, which could contribute to VCP disease pathogenesis (Tanaka et al., 2010; Kim et al., 2013; Zhang et al., 2017; Bento et al., 2018). To determine whether our VCP disease models resulted in mitochondrial dysfunction, substrate coupling control and respiratory fluxes were measured in saponin-permeabilized flight muscles (Fig. 8A; Fig. S2A) of flies at midlife (∼30 days old) when age-related defects are pronounced (Ferguson et al., 2005). Male VCP mutants developed a defect in NADH-linked respiration (N-linked) (Fig. 8B,C) from which females were protected (Fig. 8C,E; Fig. S2B). Male P134L mutants displayed defects in the primary pathways of respiratory control (Fig. 8D,F,H,J). Notably, female P134L mutants were protected from such defects (Fig. 8E,G,I,K; Fig. S2C-E). Upon addition of FCCP to facilitate maximal electron transfer, respiratory defects in VCP mutants were largely alleviated, indicating that phosphorylation rather than substrate oxidation was limiting where defects were present (Fig. 8J,K; Fig. S2F). This is in agreement with previous reports demonstrating restoration of mitochondrial respiratory control in VCP-deficient cells (Zhang et al., 2017). Taken together, these data indicate that VCP mutants develop age-related defects in mitochondrial respiratory function in a substrate and sex-specific manner.

## DISCUSSION

MSP-1 is an autosomal dominant disease that results from mutations in VCP (Watts et al., 2004). VCP mutations are also linked to other degenerative diseases, including ALS, Parkinson's disease and muscular dystrophy (Kimonis et al., 2008b; Majounie et al., 2012; Abramzon et al., 2012; Mori et al., 2013; Liewluck et al., 2014; Kazamel et al., 2015; Al-Obeidi et al., 2018). A perplexing aspect of VCP-related diseases is that VCP mutations cause a broad spectrum of seemingly unpredictable clinical phenotypes (Al-Obeidi et al., 2018). Consequently, knowledge of the specific mutation of a patient currently does little to inform any kind of personalized treatment strategy. Predicting patient outcomes based on genetic mutation will require an understanding of the etiology of the specific mutation on a deeper level. Here, we present CRISPR/Cas9-engineered *Drosophila* models that can be used to study individual VCP patient mutations on a molecular, cellular and organismal level.

To validate the usefulness of these VCP disease models, we performed a broad array of cellular and organismal assays to determine whether they mirror VCP diseases. On an organismal level, the majority of patients with MSP-1 present with muscle weakness around midlife that results in mobility impairments (Weihl et al., 2009). In agreement, we find that all of the *Drosophila* VCP disease models display progressive mobility impairments to some degree. Moreover, approximately a third of patients with VCP mutations also display memory loss, and we found that two of nine mutants (P134L and A436S) displayed memory impairments. Of the two mutants that displayed memory impairments, P134L was the most severe. Likewise, human patients with the homologous mutation (P137L) display rapidly progressive dementia (Palmio et al., 2011). Thus, on an organismal level, we find that our fly models mirror many of the symptoms observed in human patients with VCP mutations.

On a cellular level, we observed significant defects in tubular lysosomal networks, which correlated with proteinopathies, a typical pathology of MSP-1. We also observed a nuclear lamina pathology in several of the mutants that has not yet been ascribed to VCP-related diseases. However, dysmorphic nuclear shape and nuclear size defects, similar to what we have observed in our VCP models, are a hallmark feature of HGPS (De Sandre-Giovannoli et al., 2003). Similar to MSP-1, HGPS is a multisystem disorder in which patients exhibit signs of pre-mature aging (Pollex and Hegele, 2004). HGPS is caused by a truncated version of Lamin A called *progerin* that ectopically overaccumulates at the lamina and disrupts nuclear architecture (De Sandre-Giovannoli et al., 2003; Taimen et al., 2009). How VCP mutants lead to similar nuclear lamina defects remains unknown, but Lamin proteins are regulated by autophagy-dependent proteostasis (Dou et al., 2015; Borroni et al., 2018). Potentially, VCP could participate in the selective turnover of Lamin proteins, and these functions could be disrupted in VCP mutants. Dysmorphic nuclear architecture is also associated with genomic instability (Liu et al., 2005), which could compound the pleiotropic effects of VCP diseases. Exploring these mechanisms, and whether nuclear lamina defects contribute to VCP diseases, will certainly be exciting avenues to pursue.

Our study also revealed an unexpected finding of sex-specific differences in mitochondrial respiratory function; males from almost all VCP mutant genotypes displayed significant defects in NADH-linked respiration, whereas females of the same genotypes did not. NADH-linked substrates, such as pyruvate and malate, feed electrons through mitochondrial respiratory complex I, which can then be inhibited by compounds, such as rotenone (Degli Esposti, 1998). Interestingly, dysfunction of the p97/VCP system in neuroblastoma cells heightened sensitivity to complex I inhibition (Fang et al., 2015), indicating that VCP may directly or indirectly stabilize NADH-linked respiratory function. Although sex-specific differences in human patients with MSP-1 have not yet been reported, our studies suggest that at a cellular/molecular level the pathology of the disease could affect males and females differently. Moreover, although both sexes may be diagnosed proportionately, the severity of disease symptoms may vary between sexes. In support of this, our phenotypic screens revealed less severe age-dependent decline of mobility in female flies. This may be an important consideration when devising therapeutic strategies, and further understanding of the molecular mechanism behind these sex-specific differences is required.

In sum, we have generated nine knock-in VCP disease models in a genetically tractable model organism with a relatively short life cycle. Moreover, the design of our VCP CRISPR vectors make it easy to generate any VCP mutations of interest rapidly (with or without an epitope tag). We anticipate that these *Drosophila* models and CRISPR vectors will become useful tools for researchers in multiple disciplines to study VCP-related diseases under various contexts, and facilitate new discoveries about the disease etiology. Finally, our models offer the advantage of a quick and cost-effective platform for screening pharmacological and/or genetic treatment strategies that might better inform future mammalian studies and/or human clinical trials.

## MATERIALS AND METHODS

### Fly stocks

The following transgenic fly stocks were generated in this study: *Ter94-sfGFP/+*, *Ter94^P134L^-sfGFP/+*, *Ter94^R152C^-sfGFP/+*, *Ter94^R152H^-sfGFP/+*, *Ter94^A154R^-sfGFP/+*, *Ter94^R156C^-sfGFP/+*, *Ter94^R188Q^-sfGFP/+*, *Ter94^L195W^-sfGFP/+*, *Ter94^T259A^-sfGFP/+* and *Ter94^A436S^-sfGFP/+* (endogenous gene replacement for all lines). Endogenous gene replacements were generated using a scarless CRISPR strategy (Gratz et al., 2014). In brief, donor and gRNA plasmids were co-injected into vas-Cas9-expressing *Drosophila* embryos (Bloomington *Drosophila* Stock Center, 51324). The donor plasmid (pHD-Scarless; Addgene, 64703) contained the VCP transgene of interest, ∼1 kb of homologous sequence upstream and downstream of the insertion sites, and a dsRed cassette as a selection marker (Fig. 1B). Additionally, the PAM sites that were used to guide Cas9 cleavage of the endogenous genome were mutated in the donor plasmid to prevent cleavage of the transgene after insertion. The gRNA plasmid (pCFD4; Addgene, 49411) contained two gRNA sequences to direct Cas9 cleavage of sites upstream and downstream of the target genomic region to be replaced. The injection services of BestGene were used to generate transgenic flies. Transgenic animals were verified by PCR and sequencing (Fig. S1).

### Live imaging methods

Third instar larvae were dissected, and all live imaging was performed in saline solution [100 mM sodium chloride, 1 M magnesium chloride, 3 mM potassium chloride, 5 mM glucose, 1 mM sodium dihydrogen sodium monophosphate and 20 mM sodium bicarbonate (pH 7.5)]. At least three muscles from five animals were imaged for each experiment to account for the possible variations in respective muscles. Imaging was performed using a Leica DMi8 microscope with a 100× objective (1.4NA) and THUNDER imager.

### Phalloidin staining

Larval muscles from four independent animals per strain were dissected in saline solution [100 mM sodium chloride, 1 M magnesium chloride, 3 mM potassium chloride, 5 mM glucose, 1 mM sodium dihydrogen sodium monophosphate and 20 mM sodium bicarbonate (pH 7.2)]. Dissected muscles were fixed with 4% paraformaldehyde for 15 min at room temperature and washed with 1× PBS three times. The samples were incubated with 0.1% Triton X-100 solution for 15 min at room temperature, and subsequently washed three times with 1× PBS. The muscles were then incubated with Alexa Fluor 568 phalloidin (Invitrogen, A12380, 1:400) for 1 h at room temperature. After staining, samples were washed three times with 1× PBS and mounted on a glass slide using Vectashield antifade mounting medium. Samples were imaged using a Leica DMi8 microscope with a 100× objective (1.4NA) and THUNDER imager.

### Immunostaining

Adult abdominal and larval muscles were dissected in saline and fixed with 4% paraformaldehyde for 15 min or Bouin's solution for 10 min. The dissected muscles were washed with 2% PBS-Triton X-100 and incubated with rabbit polyclonal antibody Ref(2)p/p62 (rabbit pAb, Abcam, ab178440) at a 1:500 dilution, TDP-43 rabbit polyclonal antibody (pAb, Proteintech, 10782-2-Ap) at a 1:500 dilution or Lamin C mouse monoclonal antibody (mAb, Developmental Studies Hybridoma Bank, LC28.26) at a 1:100 dilution overnight at 4°C on a nutator. Subsequently, the muscles were washed three times with 2% PBS-Triton X-100 and incubated overnight at 4°C with either goat anti-rabbit-Cy5 or goat anti-mouse-Alexa 555 secondary antibodies (Invitrogen) at a 1:500 dilution. Samples were mounted on a glass slide with Vectashield antifade mounting medium and imaged using a Leica DMi8 microscope with a 100× objective (1.4NA) and THUNDER imager.

### Lysosome network analysis

Lysosome networks were analyzed using ‘skeleton’ analysis plug-ins in FIJI (National Institutes of Health). Briefly, images were converted to binary 8-bit images and then to skeleton images using the ‘Skeletonize’ plug-in. Skeleton images were then quantified using the ‘analyze skeleton’ plug-in. The number of lysosomal segments and number of junctions were scored. A lysosome segment is defined by the AnalyzeSkeleton plug-in as a section connecting two endpoints, an endpoint and a junction or two junctions. Junctions/segment was used as a parameter to quantify network integrity. Lysosomal network density was calculated in FIJI using the ‘StuartLab’ analysis plug-in, which has previously been used to quantify mitochondrial networks (Valente et al., 2017). Briefly, fluorescent images were converted to 8- or 16-bit binary images, and the area or volume of the image consumed by the signal intensity (also known as ‘footprint’) was calculated using the Stuart Lab analysis platform.

### Western blotting methods

Twelve adult flies were ground in 200 µl of 5× sample buffer [1 M Tris-HCl (pH 6.8), 1 mM DTT, 20% SDS, 60% glycerol, Bromophenol Blue] using a motorized grinder. Samples were boiled for 10 min, and proteins were resolved by SDS-PAGE on a 4-12% Bis-Tris gel (Invitrogen, Thermo Fisher Scientific), and subsequently transferred to a nitrocellulose membrane and blocked in 10% horse serum/PBS. For immunoblotting, membranes were incubated overnight at 4°C with either VCP rabbit monoclonal antibody (7F3, rabbit mAb #2649, Cell Signaling Technology), GFP polyclonal antibody (Alexa Fluor 488, Invitrogen) or actin monoclonal antibody (ACTN05) (C4, Invitrogen). Subsequently, membranes were washed three times in PBS/0.1% Tween 20 and incubated with goat anti-rabbit IgG or goat anti-mouse IgG horseradish peroxidase-conjugated secondary antibodies (Invitrogen, Thermo Fisher). Proteins were detected using an ECL chemiluminescent reagent (Bio-Rad) and imaged using a Bio-Rad GelDoc imager.

### Negative geotaxis assay

Adult male flies were collected in a 25×95 mm vial containing food. A distance of 3 cm was marked on the vial starting from the top of the food. The vial was tapped on a hard surface to bring all the flies to the bottom of the vial and the number of flies that were able to climb the 3 cm distance in 10 s were scored. Approximately 40 flies were used for each genotype in each biological replicate.

### Lifespan assay

Freshly eclosed flies were collected for each experimental strain and allowed to mate for 2 days. After mating, ∼90 males were collected for individual strains and divided into three vials containing 30 flies each to avoid overcrowding. The flies were transferred to fresh food every 2-3 days and deaths were scored at the time of transfer. The statistical analysis software OASIS was used to calculate the median lifespans and perform a log-rank test.

### Aversive phototaxis suppression assay

A custom-built T-maze that contained a light and dark chamber was used. This assay is based on the principle that flies are naturally attracted to light, except when an aversive odor (quinine hydrochloride dihydrate, MP Biomedicals) is associated with the light. For each experiment, ∼25 adult male flies were transferred from a 25×95 mm vial containing food to an empty vial, and starved for 6 h before conducting the experiment. Before each experimental trial, flies were tested to determine whether they were positively phototaxic under normal conditions. Flies were acclimated to the dark chamber for 30 s, and flies that failed to migrate towards the light chamber after 25 s were considered non-phototaxic and were censored from the experiment. For the remaining flies that were phototaxic, a filter paper soaked with quinine solution was inserted into the light chamber and 12 training trials were conducted. For each trial, flies were allowed 60 s to migrate towards the light chamber. At the end of the 60-s period, the number of flies that migrated into the light chamber were scored as ‘fail’, and the flies that remained in the dark chamber were scored as ‘pass’. Immediately after the training trials, five more test trials were conducted using the same parameters to test their short-term memory.

### Mitochondrial respiratory capacity

Oxidative phosphorylation (OXPHOS) and electron transfer capacity of permeabilized flight muscle was determined by high-resolution respirometry (Oroboros O2k; Innsbruck, Austria) as described previously (Simard et al., 2018; Axelrod et al., 2020). Briefly, 2-3 flies aged 30-35 days were sedated by cold exposure at 4°C for 7-10 min. While sedated, thorax muscle was isolated from surrounding tissue and placed into the tissue preservation solution BIOPS [50 mM K±MES, 20 mM taurine, 0.5 mM dithiothreitol, 6.56 mM MgCl_2_, 5.77 mM ATP, 15 mM phosphocreatine, 20 mM imidazole (pH 7.1) adjusted with 5 N KOH at 0°C, 10 mM Ca-EGTA buffer, 2.77 mM CaK_2_EGTA plus 7.23 mM K_2_EGTA, and 0.1 mM free calcium] (Doerrier et al., 2018). Tissues were then mechanically separated under a dissection microscope, placed into fresh BIOPS containing saponin (5 mg/ml), and gently agitated at 4°C for 20 min. Tissues were then suspended in mitochondrial respiration medium [MiR05; 110 mM sucrose, 60 mM K±lactobionate, 0.5 mM EGTA, 3 mM MgCl_2_, 20 mM taurine, 10 mM KH_2_PO_4_, 20 mM HEPES (adjusted to pH 7.1 with KOH at 37°C), and 1 g/l de-fatted bovine serum albumin] (Doerrier et al., 2018), blotted on filter paper, and weighed. Approximately 1 mg of tissue was transferred into an oxygraph chamber containing 2 ml of MiR05, oxygenated to ∼600 μM, the chamber closed, and respiration was allowed to stabilize. OXPHOS and electron transfer capacity was determined using the following concentrations of substrates, uncouplers and inhibitors: malate (2 mM), pyruvate (2.5 mM), ADP (2.5 mM), proline (5 mM), succinate (10 mM), glycerol-3-phosphate (15 mM), tetramethyl-p-phenylenediamine (TMPD, 0.5 μM), ascorbate (2 mM), carbonylcyanide-p-trifluoromethoxyphenylhydrazone (FCCP, 0.5 μM increment), rotenone (500 nM), atpenin A5 (1 μM), antimycin A (2.5 μM) and sodium azide (200 mM). The acceptor control ratio was defined as OXPHOS (P)/LEAK (L) in the presence of pyruvate and malate.

## Supplementary Material

Supplementary information
