# Supplementary material for: CRISPR/Cas9-engineered Drosophila knock-in models to study VCP diseases
Source: Dis Model Mech. 2021 Jul 12;14(7):dmm048603. doi: 10.1242/dmm.048603 (PMC8325010; doi:10.1242/dmm.048603)
Supplement: Supplementary information [file dmm-14-048603-s1.pdf]

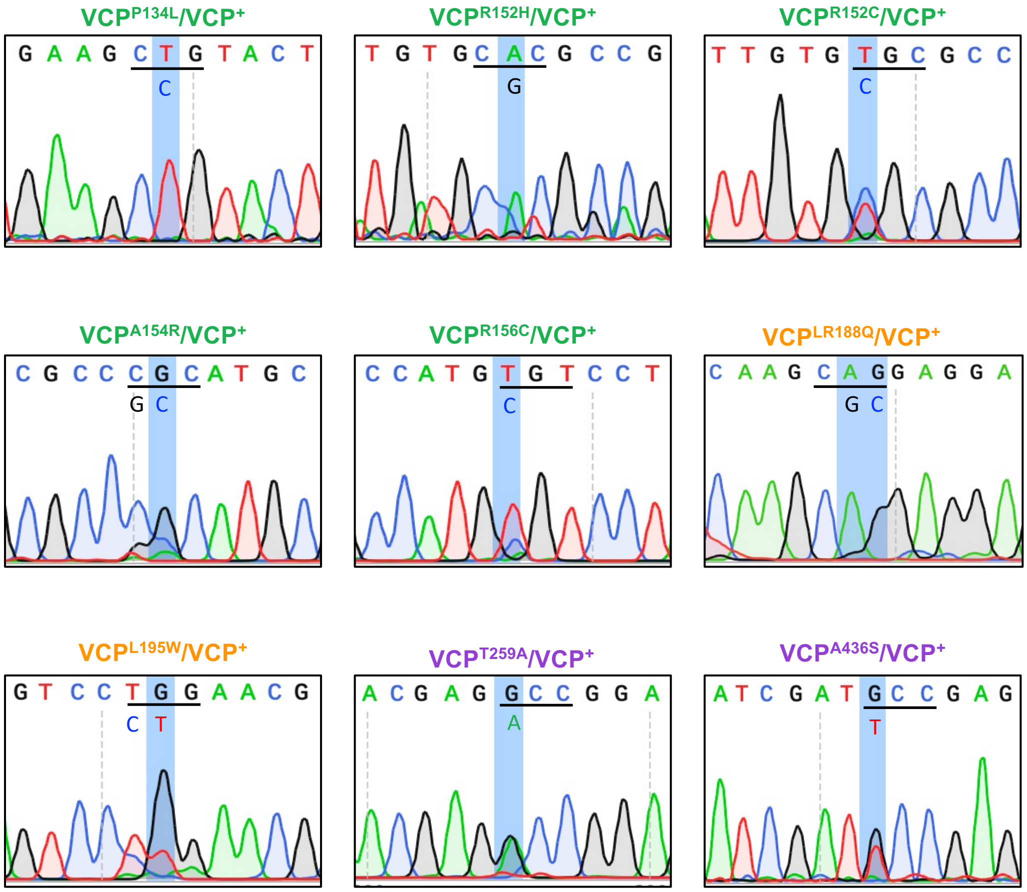

Fig. S1, related to figure 1: Sequence validation of VCP CRISPR knock-in disease models. Sequencing chromatograms for the genotypes indicated. Mutants are heterozygous; thus, in some cases two peaks are observed for the WT and mutant sequence.

Disease Models & Mechanisms • Supplementary information

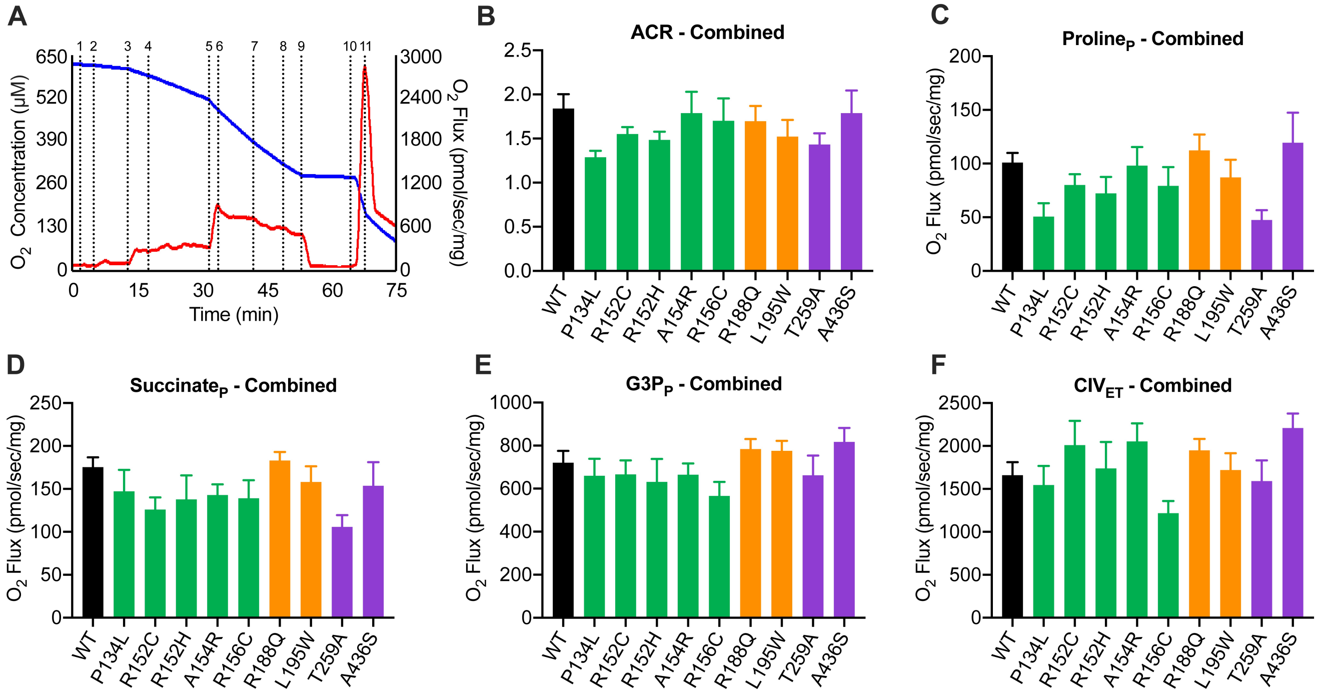

Fig. S2, related to Figure 8: Mitochondrial respiratory function in VCP mutants
A. Representative tracing of the change in oxygen concentration (blue) and flux (red) over the course of a substrate-uncoupler-inhibitor titration protocol. Numbers correspond with the following titrations: (1) pyruvate + malate, (2) ADP, (3) proline, (4) succinate, (5) glycerol-3-phosphate (G3P), (6) FCCP, (7) rotenone, (8) atpenin A5, (9) antimycin A, (10) ascorbate + TMPD, and (11) sodium azide. **B-F.** ACR (B) and respiration supported by proline (C), succinate (D), and G3P (E) in the presence of ADP (OXPHOS; P) and ascorbate + TMPD (CIV) (F) in aged male and female WT and VCP mutants. Data are presented as the mean ± SEM and were assessed by one-way ANOVA with Dunnett's multiple comparisons.

Disease Models & Mechanisms • Supplementary information
